# Variation in parasite resistance of Arctic charr, *Salvelinus alpinus*, between and within sympatric morphs

**DOI:** 10.1002/ece3.8109

**Published:** 2021-09-14

**Authors:** Anssi Karvonen, Samantha V. Beck, Skúli Skúlason, Bjarni K. Kristjánsson, Camille A. Leblanc

**Affiliations:** ^1^ Department of Biological and Environmental Science University of Jyvaskyla Jyvaskyla Finland; ^2^ Department of Aquaculture and Fish Biology Hólar University Sauðárkrókur Iceland; ^3^ Galloway Fisheries Trust Newton Stewart Scotland

**Keywords:** adaptive radiation, breeding coloration, freshwater fish ecotype, host–parasite interaction, immunogenes, speciation, trematode

## Abstract

Genetic variation in resistance against parasite infections is a predominant feature in host–parasite systems. However, mechanisms maintaining genetic polymorphism in resistance in natural host populations are generally poorly known. We explored whether differences in natural infection pressure between resource‐based morphs of Arctic charr (*Salvelinus alpinus*) have resulted in differentiation in resistance profiles. We experimentally exposed offspring of two morphs from Lake Þingvallavatn (Iceland), the pelagic planktivorous charr (“murta”) and the large benthivorous charr (“kuðungableikja”), to their common parasite, eye fluke *Diplostomum baeri*, infecting the eye humor. We found that there were no differences in resistance between the morphs, but clear differences among families within each morph. Moreover, we found suggestive evidence of resistance of offspring within families being positively correlated with the parasite load of the father, but not with that of the mother. Our results suggest that the inherited basis of parasite resistance in this system is likely to be related to variation among host individuals within each morph rather than ecological factors driving divergent resistance profiles at morph level. Overall, this may have implications for evolution of resistance through processes such as sexual selection.

## Graphical abstract

Mechanisms maintaining genetic polymorphism in resistance in natural host populations are generally poorly known. Here, we show no differences in resistance between sympatric morphs of Arctic charr, but clear differences among families within each morph. Our results suggest that the inherited basis of parasite resistance in this system is related to variation among host individuals within each morph rather than ecological factors driving divergent resistance profiles at morph level.

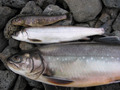

## 1 INTRODUCTION

The ability of organisms to defend against parasitic infections is central in determining their fitness. Both parasite infectivity and host resistance show marked genetic variation (Carius et al., 2001; Laine, 2007; Penczykowski et al., 2016; Seppälä et al., 2012; Susi & Laine, 2017; Vale & Little, 2009), and the outcome of infections is typically determined by complex interactions between host and parasite genotypes, *G*_Host_ × *G*_Parasite_ (Ben‐Ami et al., 2008; Carius et al., 2001; Grech et al., 2006; Susi et al., 2015). However, mechanisms maintaining such variation are not well known, but could include, for example, interactions between infections of different parasite species (Seppälä et al., 2009) and variation in infection pressure experienced by the hosts (Eizaguirre et al., 2009). Indeed, parasitism typically shows significant spatiotemporal variation, for example, because of aggregation of infected individuals or parasite intermediate host releasing the infective stages (Byers et al., 2008; Faltýnková et al., 2008; Jokela & Lively, 1995; Jousimo et al., 2014; Karvonen et al., 2005) and seasonal variation in parasite transmission (Karvonen et al., 2004; Soubeyrand et al., 2009; Taskinen et al., 1994). Thus, populations of a host species living in different microhabitats may be differently exposed to parasites. There is a growing body of literature demonstrating divergent intraspecific parasitism, particularly between morphs or ecotypes of freshwater fishes (Blais et al., 2007; Eizaguirre et al., 2011; Hablutzel et al., 2016; Karvonen, Kristjánsson, et al., 2013; Karvonen et al., 2015; Karvonen et al., 2013, 2018; Knudsen et al., 1997, 2003; Maan et al., 2008; MacColl, 2009; Natsopoulou et al., 2012; Raeymaekers et al., 2013), which on an evolutionary time scale may result in divergent evolution of resistance profiles of the populations. For example, work on threespine stickleback (*Gasterosteus aculeatus*) has shown that profiles of the major histocompatibility complex (MHC) immunogenes may readily diverge between populations exposed to different levels of parasitism (Eizaguirre et al., 2009, 2012a) and that this can take place rapidly within just few generations (Eizaguirre et al., 2012b).

Parasitism varies among individuals within populations, as some hosts are more susceptible to infections and/or become more heavily exposed to parasites than others (Karvonen et al., 2004; Shaw & Dobson, 1995). Susceptibility, or higher parasite resistance, in particular, may be genetically determined and offspring of resistant individuals may inherit these qualities. This idea is captured under the classical theory of sexual selection. For example, individuals with genes that influence higher parasite resistance may advertise their vigor to potential mates through sexual ornamentation (Hamilton & Zuk, 1982). Such ornaments are common, for example, in many species of fishes (Barber et al., 2001; Houde & Torio, 1992; Maan et al., 2006) and, although more common in males, ornaments are also often found in females (Kekäläinen et al., 2010). However, the overall evidence linking such inherited features of mother or father to the quality of their offspring in fish is unequivocal (Eilertsen et al., 2009; Figenschou et al., 2007; Huuskonen et al., 2009; Jacob et al., 2007; Janhunen et al., 2011; Janhunen et al., 2011; Polacik & Reichard, 2009; Rideout et al., 2004; Rudolfsen et al., 2005; Wedekind et al., 2001, 2008). For example, relatively high maternal effects following allocation of resources to eggs are often important in this respect (Janhunen et al., 2010; Johnston et al., 2007). Similar to other life‐history traits, effects of parental genetic background on parasite resistance of offspring are also unclear. For example, egg survival in whitefish (*Coregonus* sp.) during bacterial infection has been shown to be positively associated with the breeding ornamentation of the males, suggesting inherited effects (Wedekind et al., 2001). Further, experimental exposures in the same system showed that the importance of the maternal and paternal effects depended on the bacterial dosage (von Siebenthal et al., 2009). In contrast, male roach (*Rutilus rutilus*) with lower parasite burdens produced offspring with lower survival (Kortet et al., 2004) whereas in Arctic charr (*Salvelinus alpinus*) offspring parasite resistance varied between females sired by the same male (Kortet et al., 2017). In the present work, we tested whether parasite resistance differed between well‐diverged sympatric morphs, and families within the morphs, of Arctic charr from Lake Þingvallavatn, Iceland.

**FIGURE 1:**
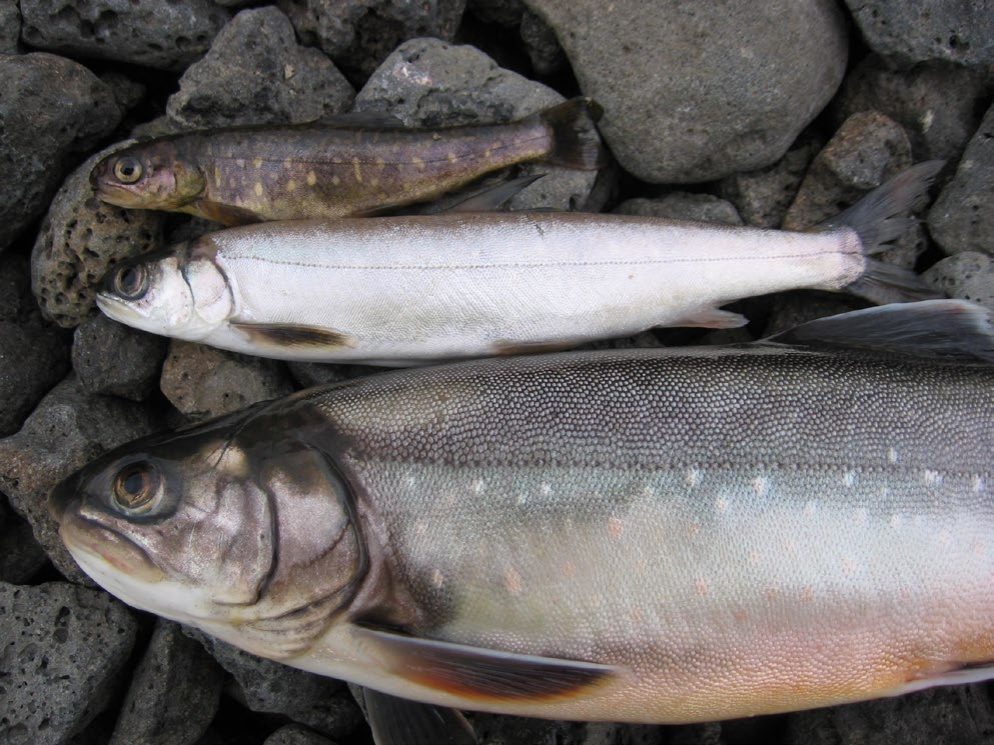
Lake Þingvallavatn in Iceland has four morphs of Arctic charr (*Salvelinus alpinus*), each with a distinct phenotype, habitat specialization, and life‐history characteristics. The small benthivorous morph (top) is found in the littoral zone and feeds on benthic invertebrates. The pelagic planktivorous morph, “murta” (middle), is the most abundant morph in the lake. Larger individuals of the piscivorous morph (bottom) feed on sticklebacks and small charr. The fourth morph, the large benthivorous morph (Figure 2, bottom), inhabits the littoral zone of the lake and feeds predominantly on benthic invertebrates, similar to the small benthivorous morph

**FIGURE 2:**
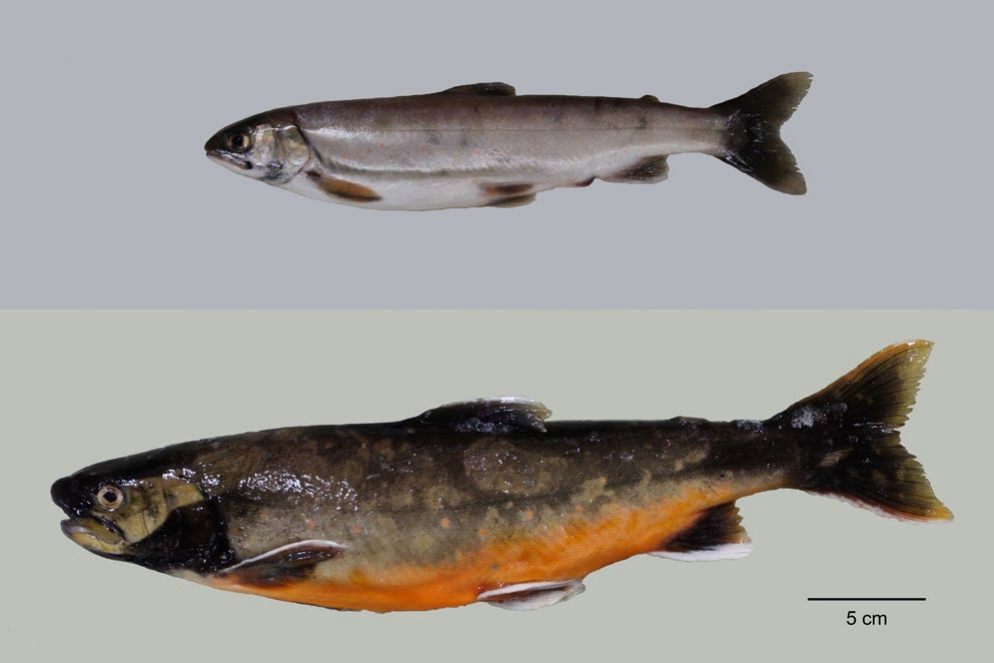
Pelagic planktivorous (top) and large benthivorous (bottom) morphs of Arctic charr (*Salvelinus alpinus*) from Lake Þingvallavatn, investigated for infections of *Diplostomum baeri* in the eye humor in the present study

Arctic charr is a salmonid fish species, which has colonized a number of lakes in the Arctic and Subarctic following the last glacial period. It has become one of the hallmark species of rapid adaptive radiation and speciation among freshwater fishes with several northern lake systems now harboring two or more sympatric or parapatric morphs (Gíslason et al., 1999; Jónsson & Skúlason, 2000; Knudsen et al., 1997; Skúlason et al., 1999). These morphs typically show specialized morphological and ecological features including differences in habitats, feeding, life‐history traits, and reproduction (Skúlason et al., 1999). The largest lake in Iceland, Þingvallavatn, currently has four distinct morphs of Arctic charr (Figures 1 and 2), each with a specialized habitat and timing of reproduction (Jónsson et al., 1988; Malmquist et al., 1992; Sandlund et al., 1992; Skúlason et al., 1989). The two benthic morphs, the large and small benthivorous charr (Figures 1 and 2), inhabit littoral zones of the lake feeding mainly on benthic invertebrates such as snails (Malmquist et al., 1992). The most abundant morph is the pelagic planktivorous charr “murta” (Figure 2), which feeds mainly on zooplankton already from early age (Sandlund et al., 1992). The fourth morph is the large piscivorous charr (Figure 1) that after reaching a certain size feeds mainly on stickleback, but also to a lesser degree on smaller charr (Malmquist et al., 1992). Due to the differences in habitat and feeding ecology, these morphs are differentially exposed to parasites (Frandsen et al., 1989). For example, parasite communities of the benthic morphs are dominated by trematodes transmitted from benthic snails. On the other hand, the pelagic morphs harbor fewer trematodes, but higher numbers of cestodes, transmitted via zooplankton (Frandsen et al., 1989).

Here, we explored whether the resistance profiles differ within and between the large benthivorous and the pelagic planktivorous charr from Þingvallavatn. We experimentally exposed a number of juveniles belonging to different families of both morphs to their common trematode parasite, *Diplostomum baeri*. The parasite is transmitted to fish as free‐living clonal larvae (cercariae) that are produced asexually in the snail intermediate hosts. Cercariae enter the fish and migrate to the eye humor, where they develop to metacercariae. We were particularly interested if the differences in the degree of exposure between the morphs in the wild (Frandsen et al., 1989) have resulted in differentiation in their resistance to the infection. Further, we compared the magnitude of variation in resistance between the morphs and among families within morphs and contrasted the parasite numbers that the parental fish had acquired in the wild to that of their offspring in the experimental exposure. We expected (1) that the benthivorous charr would show higher resistance to infection as it experiences higher infection pressure from this parasite in the wild, compared with the planktivorous charr; and (2) that parasite numbers of the offspring families would be positively correlated with parasite numbers of the parental fish.

## 2 MATERIALS AND METHODS

Wild mature Arctic charr were caught using gill nets in Lake Þingvallavatn during the time of spawning. Seven females and five males of the large benthivorous charr were caught on the 6th of August 2015 at Ólafsdráttur (N 64 13.927 W 021 03.12). Eight females and five males of the planktivorous charr were caught on the 9th of October 2015 in the bay North of the Mjóanes peninsula. Families were created by mixing eggs and sperm of each morph by pairing one female with one male, with some of the males used for two females. Fertilized eggs were water hardened in the field before transport to Verið, Hólar University rearing facilities in Sauðárkrókur, North Iceland. Until first feeding (approximately 5 months from fertilization), embryos were raised in family groups in mesh cages maintained in darkness in a vertical shelf incubator, as described in Beck et al. (2019). Before the onset of first feeding, offspring were transferred to 20‐L tanks with continuous water flow (5.16°C ± *SD* 0.4) and fed with commercial aquaculture fish food. All tanks received water from the same water source and were located in the same room. The tanks were rotated regularly and randomly to minimize possible tank effects. Fish were maintained in these conditions until August 2016, when they were 10–12 months old.

Before the parasite exposure, water temperature in the rearing tanks was slowly brought up to 15°C, to ensure infectivity of the parasite (Chappell et al., 1994; Karvonen et al., 2006). Cercariae of *D. baeri* were produced from 23 naturally infected *Lymnaea peregra* snails collected from nearby lakes. Parasites were initially identified from cercarial morphology, and the site of infection in the eye humor of fish was confirmed using pre‐trial infections. It should be noted, however, that “*Diplostomum baeri*” is a species complex that includes several species, identifiable using molecular analysis (Blasco‐Costa et al., 2014). We used the species name “*D. baeri*” in this study to refer to infections in the eye humor, but recognize that more than one species of the species complex may have been present. Snails were allowed to produce cercariae for 3 hr at 20°C and the suspensions from the snails were pooled. Cercarial density in the pooled suspension was determined from 10 × 1 ml samples. A maximum number of 40 fish from each morph‐family combination (Table 1) were individually exposed to the parasite for 30 min in containers with 0.5 L of water (15°C) and 150 parasite cercariae. After the exposure, fish were maintained in replicated containers for a minimum of 24 hr to allow parasite establishment (Louhi et al., 2015). No mortality of fish took place during the temperature increase, or during or after the parasite exposure.

**TABLE 1:** Number of fish, mean total body length (±*SE*), and range in number of parasites (min‐max) in families of large benthivorous and pelagic planktivorous morphs of Arctic charr (*Salvelinus alpinus*) from Lake Þingvallavatn, exposed experimentally to the trematode *Diplostomum baeri*

Morph	Family	*N* fish exposed	Mean length (mm) ± *SE*	Range in number of parasites
Benthic	116	39	56.28 ± 0.42	11–48
	118	40	53.50 ± 0.42	7–40
	119	37	45.43 ± 0.50	9–37
	122	40	54.80 ± 0.46	13–46
	123	37	41.59 ± 0.55	7–44
	124	39	48.03 ± 0.53	10–37
	128	30	54.37 ± 0.62	8–56
Pelagic	2	15	53.13 ± 0.97	14–32
	3	39	49.46 ± 0.34	13–40
	4	4	46.25 ± 0.63	22–57
	5	40	46.55 ± 0.39	4–40
	8	40	51.28 ± 0.40	13–48
	9	40	52.78 ± 0.42	8–45
	12	6	48.00 ± 1.67	6–29
	18	19	48.79 ± 0.59	14–41

All fish were subsequently euthanized with an overdose (600 ppm) of 2‐phenoxyethanol, measured for total length and dissected for infections in the eye humor. Eyes of all parent fish were also dissected for infections in the humor. There were no infections in eye lenses in any of the fish. Data on the experimental infection were analyzed using a mixed‐model ANCOVA with fish morph as a fixed factor, family nested under fish morph as a random factor, and fish length as a covariate. Parasite numbers of the parents were analyzed using GLM with a negative binomial probability distribution and a log‐link function, and fish morph and gender as factors, and fish length as a covariate. To contrast infections in the parents with those of the offspring, parasite numbers of the planktivorous and benthivorous females and males (standardized residuals from length) separately were plotted against the predicted mean number of parasites in the offspring families (predictions from the ANCOVA model), and the anticipated positive relationships were analyzed using one‐tailed Spearman correlations. Furthermore, to test for overall positive pattern across females and males of both morphs, Fisher's meta‐analysis (Sokal & Rohlf, 1998) was used to combine the gender‐specific correlations. This analysis sums the ln‐transformed one‐tailed p‐values of each correlation (2, 1 per morph; in case of a negative correlation, the p‐value for a positive association was calculated as 1‐p), multiplies it by −2, and compares the resulting value to a chi‐square distribution with *df* = 4 (2 × the number of tests). All tests were conducted using IBM SPSS 26 package. All experimental procedures conformed to the legislation of Iceland and were conducted under permission from the site of research (Verið‐ Hólar University rearing facilities).

## 3 RESULTS

**FIGURE 3:**
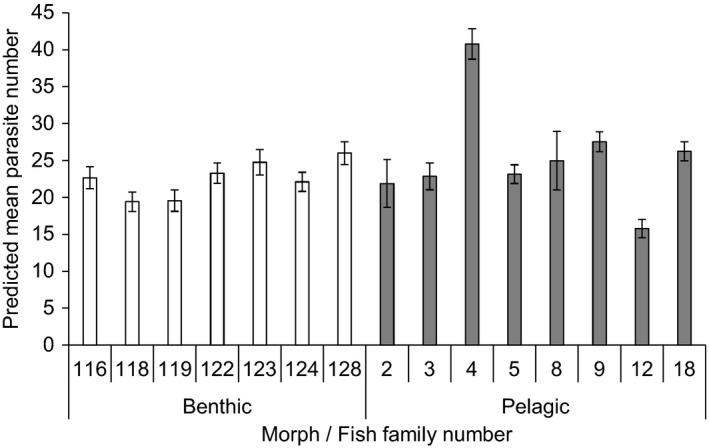
Predicted mean number (±*SE*) of *Diplostomum baeri* in eye humor of families of large benthivorous (open bars) and pelagic planktivorous (filled bars) morphs of Arctic charr (*Salvelinus alpinus*) from Lake Þingvallavatn following an experimental exposure to the parasite. Predictions are from an ANCOVA model using fish morph as a fixed factor, fish family nested within morph as a random factor, and fish length as a covariate

The mean number of *D. baeri* parasites differed among the families of the benthivorous and the planktivorous charr following the experimental exposure (nested ANCOVA: *F*_13,449_ = 4.336, *p* < .001), but overall they did not differ between the charr morphs (predicted mean number of parasites ± *SE* = 22.54 ± 0.49 and 25.40 ± 0.79 for the benthivorous and planktivorous charr, respectively; *F*_1,16.08_ = 2.880, *p* = .109; Figure 3). The effect of fish length was also not significant (*F*_1,449_ = 2.157, *p* = .143). Exclusion of the two planktivorous charr families with lower sample sizes (families 4 and 12; Table 1) did not change the results. In two of the five pairs of females sired by the same male, offspring parasite numbers differed between the families (*t* test: *t*_68_ = 3.123, *p* = .003 (benthivorous charr families 118 and 128); *t*_42_ = 3.745, *p* < .001 (planktivorous charr families 4 and 5)), suggesting differences in resistance of the offspring of the same male depending on the female.

There was a difference in mean parasite numbers between the large benthivorous and the planktivorous charr parents (GLM: Wald = 8.225, *p* = .004 (morph)), but no differences between males and females (Wald = 0.007, *p* = .932 (sex), Wald = 0.002, *p* = .967 (morph × sex)). Mean parasite numbers (±SE) in the eyes of the parent fish were 530.4 ± 82.3 (range 328–819) and 520.8 ± 37.1 (394–604) for females and males of the benthivorous charr, respectively, and 164.1 ± 19.9 (107–271) and 155.8 ± 52.0 (33–323) for the females and males of the planktivorous charr, respectively.

**FIGURE 4:**
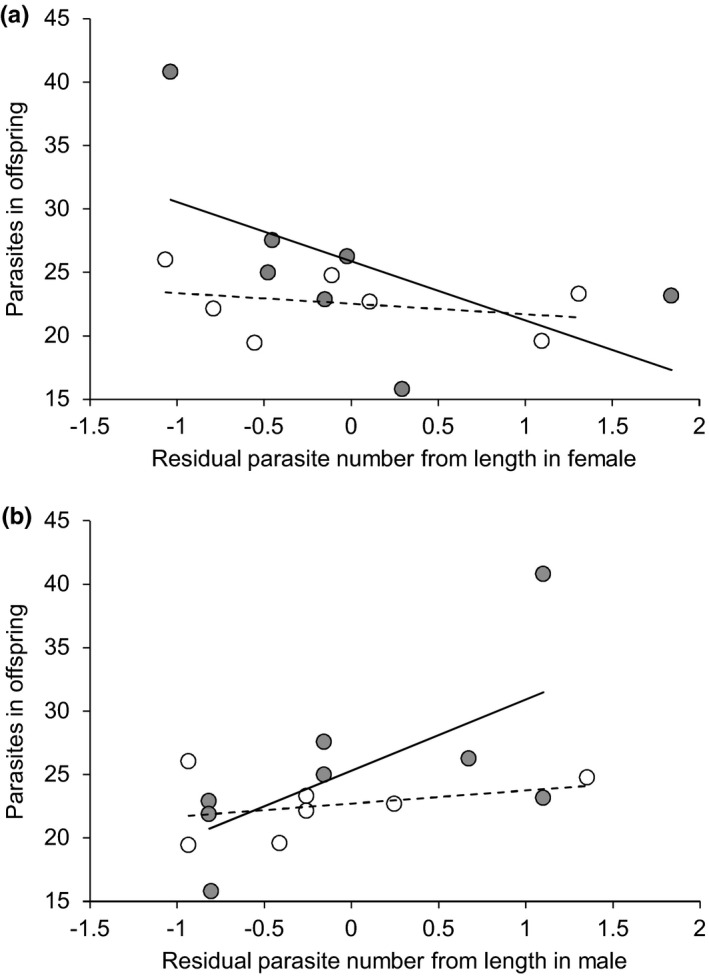
Relationships between the predicted numbers of *Diplostomum baeri* parasites in the offspring families of large benthivorous (open dots) and pelagic planktivorous (filled dots) morphs of Arctic charr from Lake Þingvallavatn (*y*‐axis, predictions from an ANCOVA model) and the standardized residual parasite numbers from length of the wild‐caught parent females (a) and males (b). The fitted lines (dashed line for benthivorous morph, solid line for pelagic morph) are linear regressions indicating the direction of the relationships

When contrasting parasite numbers of the parents with those of their offspring, the relationship was positive for planktivorous males (one‐tailed Spearman correlation: *r* = 0.691, *n* = 8, *p* = .029), and positive, but not significant, for benthivorous males (*r* = 0.291, *n* = 7, *p* = .263; Figure 4), suggesting that offspring of planktivorous males with higher parasite numbers were more susceptible to infection. The combined relationship between the morphs, however, was also significant (*χ* ^2^ = 9.75, *df* = 4, *p* = .045), suggesting positive overall relationship between parasite numbers of the offspring and those of the males. Averaging parasite numbers across families with the repeated use of males did not change the direction of the relationships (planktivorous males: *r* = 0.800, *n* = 5, *p* = .052; combined test for a positive relationship: *χ* ^2^ = 8.24, *df* = 4, *p* = .083). However, no such positive relationship was observed for females (*χ* ^2^ = 0.96, *df* = 4, *p* = .916; Figure 4).

## 4 DISCUSSION

Ecological differences in habitat use and feeding ecology of freshwater fishes can result in differences in infection pressures from parasites (Karvonen et al., 2018; Knudsen et al., 1997; Maan et al., 2008; MacColl, 2009; Raeymaekers et al., 2013), which can drive divergent evolution of resistance profiles in host populations (Eizaguirre et al., 2012a, 2012b). Typically, this is seen as higher allocation to defense in populations experiencing higher infection levels (Kalbe & Kurtz, 2006; Piecyk et al., 2019; Scharsack et al., 2016; Weber et al., 2017). We investigated resistance in two sympatric morphs of Arctic charr from a large Icelandic lake, Þingvallavatn, where the morphs experience different levels of infections from their common parasite, *Diplostomum baeri* (Frandsen et al., 1989). We experimentally tested if the anticipated differences in resistance were better explained at morph or family level. Contrary to our expectation, we found no difference in resistance between the benthic and pelagic morphs. Instead, we observed significant variation in resistance among the families within the morphs. Furthermore, the results suggest that this variation was positively correlated with the parasite numbers of the father, but not the mother, suggesting that offspring could benefit from inherited effects from their fathers. This result was evident particularly in the planktivorous morph, but also as an overall relationship calculated across the morphs.

Differences in feeding ecology of the large benthivorous and the planktivorous charr in Þingvallavatn (Malmquist et al., 1992) are likely the main reasons for their different parasite infections (Frandsen et al., 1989). While the benthivorous charr feeds predominately on benthic invertebrates such as snails, the planktivorous charr feeds on zooplankton (Malmquist et al., 1992). It is the spatial overlap of the benthivorous charr with the snails, intermediate hosts of the *Diplostomum* trematodes, which is likely to result in their higher exposure. Indeed, we found a significant difference in numbers of *Diplostomum* in the parental fish, with the benthivorous charr harboring, on average, over three times higher numbers compared to the planktivorous charr. This is in accordance with the earlier results in Frandsen et al. (1989) on parasite fauna in these morphs. However, the fact that we did not find a difference in resistance between the morphs progeny may be due to the planktivorous charr nevertheless becoming exposed to the parasite to a relatively high degree. For example, evidence suggesting divergent resistance profiles between lake and river ecotypes of threespine stickleback (Kalbe & Kurtz, 2006) typically come from systems where differences in infections between the ecotypes are substantial, with the river ecotypes showing no or very low infection (Kalbe et al. (2002); see also Eizaguirre et al. (2012a); Karvonen et al. (2015)). Thus, it is possible that potential divergent evolution in resistance in the present system would require stronger differentiation in infection rates, particularly so that one of the populations would be nearly free from infection. At higher infection levels, on the other hand, possible differences in resistance, if any, could be detected on an individual rather than population level.

Indeed, we found that the offspring of the less‐infected fathers tended to have higher parasite resistance, while no such relationship was observed for the mothers. The positive relationship for the fathers suggests that the offspring could benefit from resistance of their fathers in accordance with the “good genes” hypothesis of sexual selection (Hamilton & Zuk, 1982). Overall, the evidence linking male quality and the quality of their offspring in Arctic charr is currently equivocal (see Introduction for examples of other systems). For example, some studies have suggested a positive link between male quality and characteristics of their offspring (Eilertsen et al., 2009; Masvaer et al., 2004; Pakkasmaa et al., 2006), while others have not found such a relationship (Figenschou et al., 2007; Janhunen, Kekäläinen, et al., 2011; Janhunen, Peuhkuri, et al., 2011). Moreover, in a study exploring the effect of parental background on resistance of charr against *Diplostomum* spp. infecting eye lenses, parasite taxa closely related to *D. baeri*, Kortet et al. (2017) found that resistance at family level was better explained by the female than the male effects. However, the study used aquaculture brood fish and hence did not explore interactions between parental infections and those of the offspring.

The present data from offspring of wild parent fish suggest that parasite resistance could potentially be influenced by the infection status of the father. It should be noted, however, that some families of the planktivorous morph had relatively low sample sizes and the observed patterns were driven to some extent by such data points. Some of the males were also used to fertilize eggs from two females, although the direction of the patterns remained after averaging between these families. Moreover, interindividual differences in parasite numbers in wild‐caught hosts can result from both susceptibility and exposure (Karvonen et al., 2004; Shaw & Dobson, 1995), and therefore, parasite numbers of the parental fish do not necessarily reflect resistance alone. Thus, the present evidence linking parasite numbers of the parents and those of the offspring should be interpreted as suggestive. Interestingly, in some of the cases where the same male was used twice, the resistance of the offspring differed between the two families (see also Kortet et al. (2017)). This suggests that not all females would benefit from mating with a male with a lower level of infection, but the overall resistance could depend on the compatibility of the male and female (Huuskonen et al., 2009; Kekäläinen et al., 2010). In many of the above examples on Arctic charr, male quality is also linked with dominance or brightness of their breeding coloration (see also Skarstein and Folstad (1996); Johansen et al. (2019)). Although we did not score such variables, their possible connection with parasite resistance in parents and offspring in this system warrants interesting further investigations.

## CONFLICT OF INTEREST

The authors have no conflict of interest to declare.

## AUTHOR CONTRIBUTION

**Anssi Karvonen:** Conceptualization (equal); Data curation (lead); Formal analysis (lead); Project administration (equal); Visualization (lead); Writing‐original draft (lead); Writing‐review & editing (lead). **Samantha V. Beck:** Conceptualization (equal); Formal analysis (equal); Resources (equal); Writing‐review & editing (equal). **Skúli Skúlason:** Conceptualization (equal); Project administration (equal); Resources (equal); Writing‐review & editing (equal). **Bjarni K. Kristjánsson:** Conceptualization (equal); Project administration (equal); Resources (equal); Writing‐review & editing (equal). **Camille A. Leblanc:** Conceptualization (equal); Formal analysis (equal); Project administration (equal); Resources (equal); Writing‐review & editing (equal).

## Data Availability

Data are deposited in the Dryad Digital Repository: https://doi.org/10.5061/dryad.bvq83bk95.
